# Linking Negative Views of Aging to Cognitive Function: The Dopaminergic Theory

**DOI:** 10.1093/geroni/igaa057.2008

**Published:** 2020-12-16

**Authors:** Deidre Robertson, Arun Bokde

**Affiliations:** 1 Economic and Social Research Institute, Dublin, Ireland; 2 Trinity College Dublin, Dublin, Dublin, Ireland

## Abstract

Negative views on aging are known to impair cognition, in both the short and long term, but it is not fully understood why. This paper proposes that negative views impair cognition through dysregulation of reward-seeking behaviours and downregulation of the dopaminergic system. In a preliminary study to test this, 70 older adults were exposed to implicit positive and negative views on aging in a within-subject design carried out over 2 weeks, and then played a computerised game that involved deciding whether to expend effort to gain a reward. Over 216 trials, when participants were exposed to negative views, they were less likely to choose the effort-for-reward option. This effect was amplified at higher levels of effort. The paper will discuss how the dopaminergic theory ties into stereotype embodiment and other theories, and why it may explain previous findings that negative views of aging impair specific cognitive functions more than others.

